# Supplementary material for: Effect of X-ray free-electron laser-induced shockwaves on haemoglobin microcrystals delivered in a liquid jet
Source: Nat Commun. 2021 Mar 15;12:1672. doi: 10.1038/s41467-021-21819-8 (PMC7960726; doi:10.1038/s41467-021-21819-8)
Supplement: Supplementary file 1 — Supplementary Information [file 41467_2021_21819_MOESM1_ESM.pdf]

Supplementary Information:

Effect of X-ray free-electron laser-induced shockwaves on haemoglobin microcrystals delivered in a liquid jet

M.L. Grünbein *et al.*

Supplementary Figures

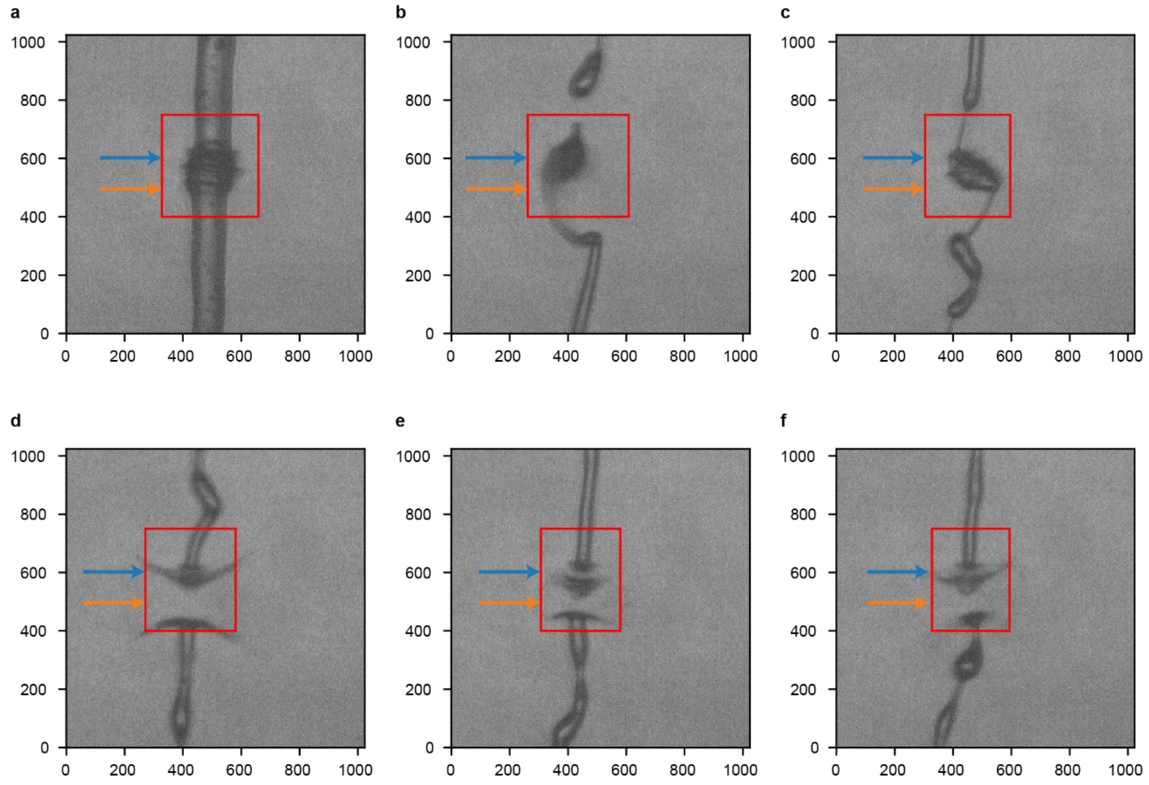

Supplementary Figure 1. **Femtosecond snapshot imaging of the jet. a-c** Exemplary jet images of data not considered in the shock analysis because of ambiguous shock wave propagation. **a** Temporary much larger diameter jet, e.g. due to resolution of a temporary clog. Moreover, no clear pump-induced gap is visible. **b** Jet in the midst of breaking up. Propagation of the shock wave to the site of probing is ambiguous due to the break-up into droplets. **c** Jet in the midst of breaking up, pump-induced explosion not visible. Most likely, no shock wave was launched. **d-f** Exemplary images of data considered in the shock analysis. **a-f** The vertical positions of pump and probe pulses are indicated by the orange and blue arrow, respectively.

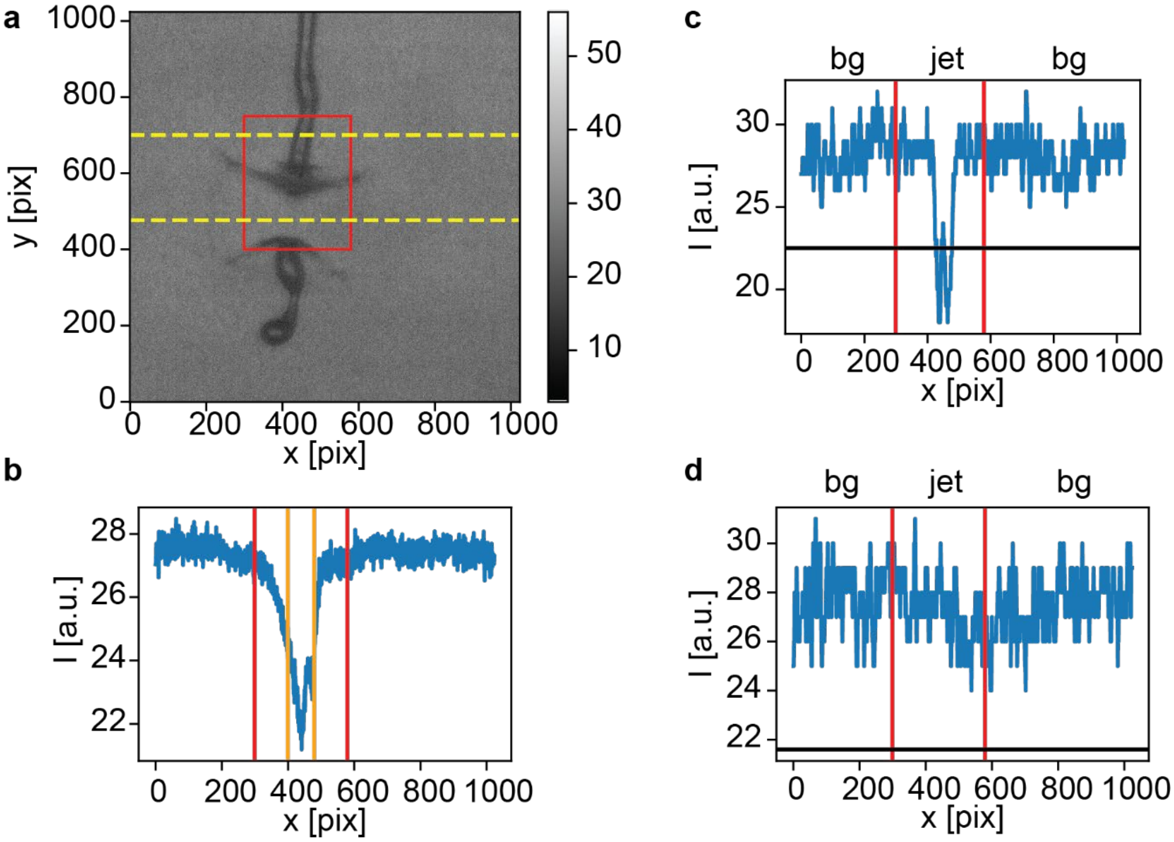

Supplementary Figure 2. **Classifying hits based on the femtosecond snapshot images.** Hits of the jet were characterized to identify pump-probe shots in which a well-defined shock wave has been launched by the pump pulse that affects the jet segment interrogated by the probe pulse. Such jets have neither abnormal shape (e.g. very skewed), nor have broken up into droplets which prohibits shock wave propagation. The latter means that jet images must show 1 or 2 gaps in the jet (indicating being hit by the pump or pump and probe pulse) and the gaps must be located in the X-ray interaction region. **a** Snapshot of the jet a few nanoseconds after interaction with the probe pulse. The gap due to pump pulse interaction is clearly visible. The red rectangle marks the region of interest in which gaps within the jet are analysed. **b** Projection of the mean pixel intensity onto the x-axis. The jet (orange boundaries) is defined as the 'full-width half-minimum' of the intensity scan. Adding 100 pixels left and right to the centre (red boundaries) delivers the region of interest in x in which the jet is analysed. **c**, **d** Line scan intensities of the 5x5 median filtered image at $y = 700$ pix and $y = 477$ pix, indicated as dashed yellow lines in (**a**). By comparing the minimum intensity of the image in the background region (bg) to the minimum intensity inside the region of interest containing the jet (confined by the red boundaries), positions along the jet axis y can be identified in which a jet is present (**c**) or which represents a gap (**d**). The black horizontal line indicates the threshold (corresponding to 90 % of the minimum pixel intensity of the background regions) which pixel intensities within the jet region have to cross in order to be identified as a jet segment.

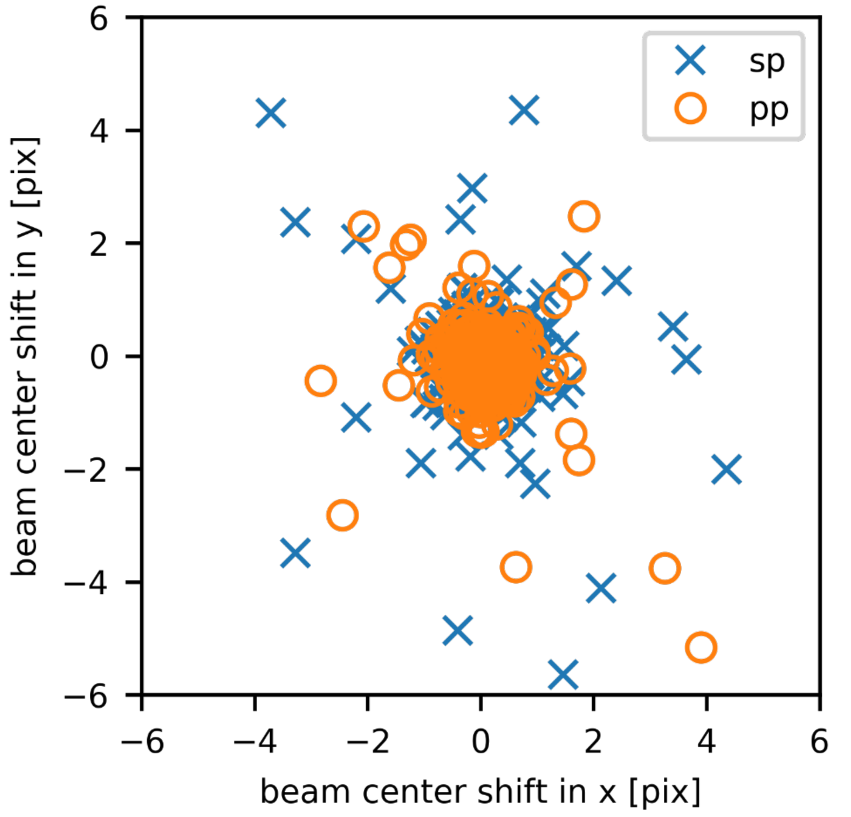

Supplementary Figure 3. Direct beam coordinates. The coordinates of the direct beam position as determined from the indexed diffraction patterns. The positions in the single-pulse and pump-probe data are shown in blue and orange, respectively. The pixel size is 110 µm.

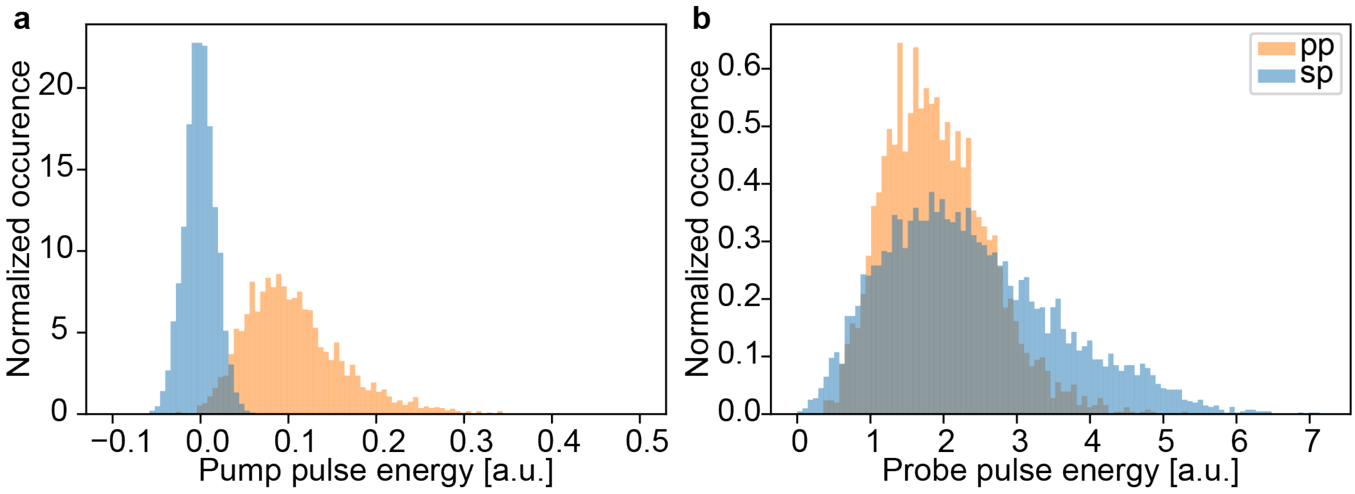

Supplementary Figure 4. **Distribution of pump and probe pulse energies in each data set. a** Histograms of pump-pulse energies measured for each shot by the unmasked diode in the pump-probe (orange) and single-pulse (blue) data set. As expected, the pump-pulse is off (pulse energy centred around 0) in the single-pulse data set. **b** Histograms of probe-pulse energies measured for each shot by the diode covered with the same thickness of iron foil as the CSPAD detector in the pump-probe (orange) and single-pulse (blue) data set. The probe-pulse energies in the single-pulse and pump-probe data are similar but not identical.

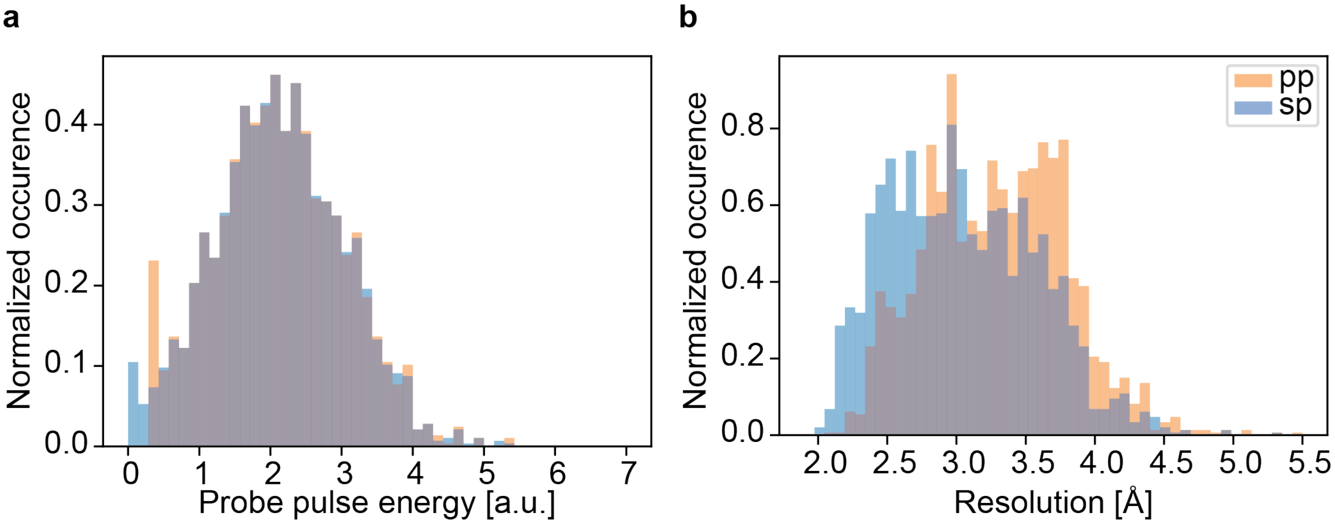

Supplementary Figure 5. **Equalizing probe pulse energy distributions of the pump-probe and single-pulse data sets, respectively. a** Post probe-pulse-energy-equalization histograms of probe-pulse energies measured for each shot by the masked diode in the pump-probe (orange) and single-pulse (blue) data set. **b** Post probe-pulse-energy-equalization histograms of the resolution of indexed hits in the pump-probe (orange) and single-pulse reference (blue) data set. The median resolution is 3.3 Å in the pump-probe case and 3.0 Å for the single-pulse reference data. The resolution limit corresponds to the highest resolution value of an indexed diffraction peak with a signal-to-noise ratio (SNR) of SNR ≥ 4. **a**,**b** A subset of N=2000 data points was sampled from each data set (with replacement) to obtain a target Gaussian probe-pulse energy distribution with centre μ=2.15 and standard deviation σ=0.94.

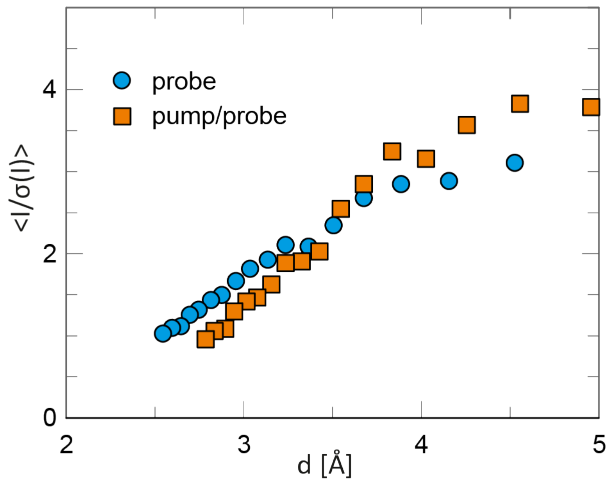

Supplementary Figure 6. **Average integrated diffraction intensity as a function of resolution shell of the pump-probe and the single-pulse data sets.** The average signal to noise ratio, I/σ(I), of the integrated diffraction intensities is plotted as a function of resolution for the pump-probe (orange) and single-pulse (blue) data set. In the pump-probe data, the signal strength and quality is worse at high resolution than for the single-pulse data. This, as well as the increase at low resolution is indicative of overall disorder.

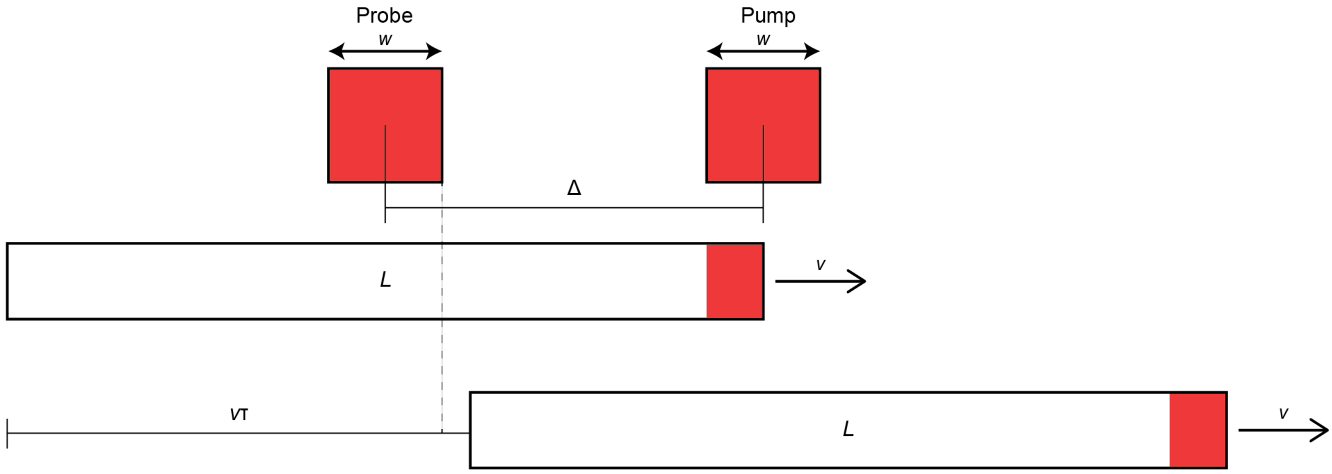

Supplementary Figure 7. Possibility of hitting the same crystal with both pulses. Experimental parameters must be chosen such that a given crystal cannot be hit (and compromised) by the pump beam prior to being interrogated by the probe beam. Consider the special case of a crystal having its leading edge centred on the pump beam as that pulse arrives (middle segment of diagram) and ask where the trailing edge of that crystal will be when the probe beam arrives (lower segment). The relevant parameters are the crystal length *L* (along the jet axis), the vertical offset Δ between the pump and probe pulse interaction regions, the beam diameter *w* of pump and probe, the time delay τ between pump and probe and the jet speed *v*. In our case *L*=~10 µm, Δ=5 µm, *w*=1.5 µm, *v*=50 m/s and τ=0.1225 µs. With these parameters, a crystal that is interrogated by the pump beam centre at its front end, will have passed 1 µm beyond the probe beam centre upon probe pulse arrival. Only a crystal that is hit by the wings of the pump pulse can be interrogated by the wings of the probe beam at its very rear end.

Specifically, this means that for $x \leq 0.375$ µm the crystal is interrogated by both pulses (x denotes the distance between the leading end of the crystal and the upstream edge of the pump beam, see Supplementary Figure 8, Supplementary Software 1), implying that the likelihood of hitting the same crystal with both pulses is <4 %. However, in such a double hit scenario, the crystal can only be interrogated by the outmost wings of both pulses, making it unlikely to lead to sufficiently strong diffraction required for being detected as a crystal hit. For $x = 0$ µm (crystal interrogated by the outmost wings of the pump pulse), the crystal is located ~0.4 µm downstream of the probe beam centre, thus being interrogated only by the wings of the probe pulse (which has a beam radius (FWHM) of $w/2$=0.75 µm). For larger x (initial position of the crystal further downstream) the effective probing intensity decreases since the crystal is further and further away from the probe beam centre when the probe pulse arrives.

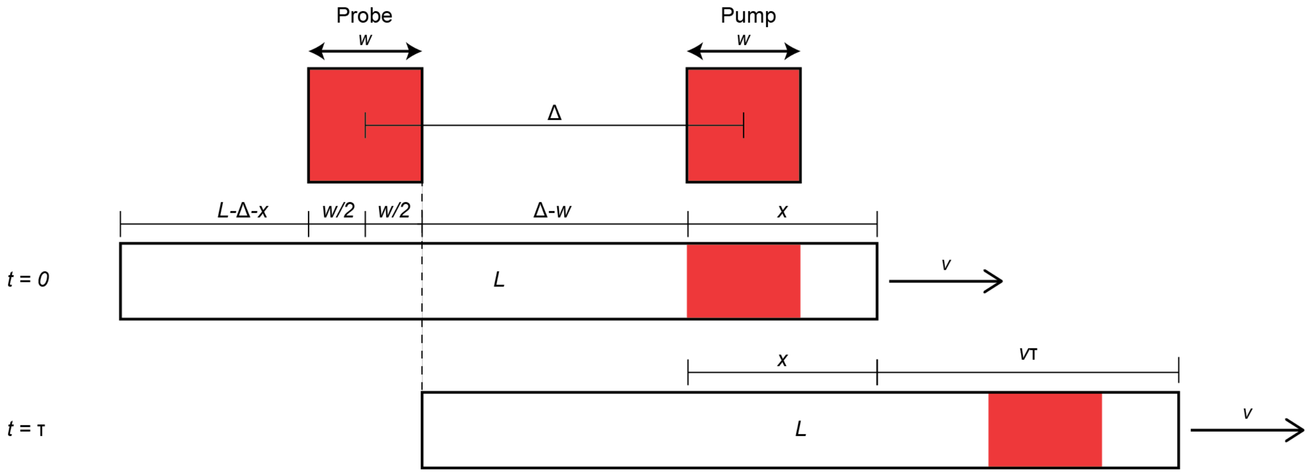

Supplementary Figure 8. General case of the probability of XFEL pump-probe dual hits on single crystals. The special case of Supplementary Figure 7 can be easily extended to compute the probability of a double hit for chosen experimental parameters. Whether the very same crystal can be interrogated by both pump and probe pulse depends on the crystal length L (along the jet axis), the vertical offset Δ between pump and probe pulse interaction region, the beam diameter w of pump and probe, the time delay τ between pump and probe and jet speed v. Consider that the leading edge of the crystal has passed a distance x beyond the upstream edge of the pump beam as the pump pulse arrives time t=0. At t=τ (arrival of the probe pulse), the trailing edge of the crystal has passed beyond the downstream edge of the probe beam and is not intersected by the probe beam anymore if $L - \Delta - x + w/2 - v\tau < -w/2$ or $x > L - \Delta + w - v\tau$. Since crystals arrive stochastically with respect to pump pulses, all values of x are equally likely and the overall probability of a double hit is then immediately calculated.

A spreadsheet is provided (Supplementary Software 1) to visualize the probability of pump-probe dual hits as a function of crystal length for various jet speeds.

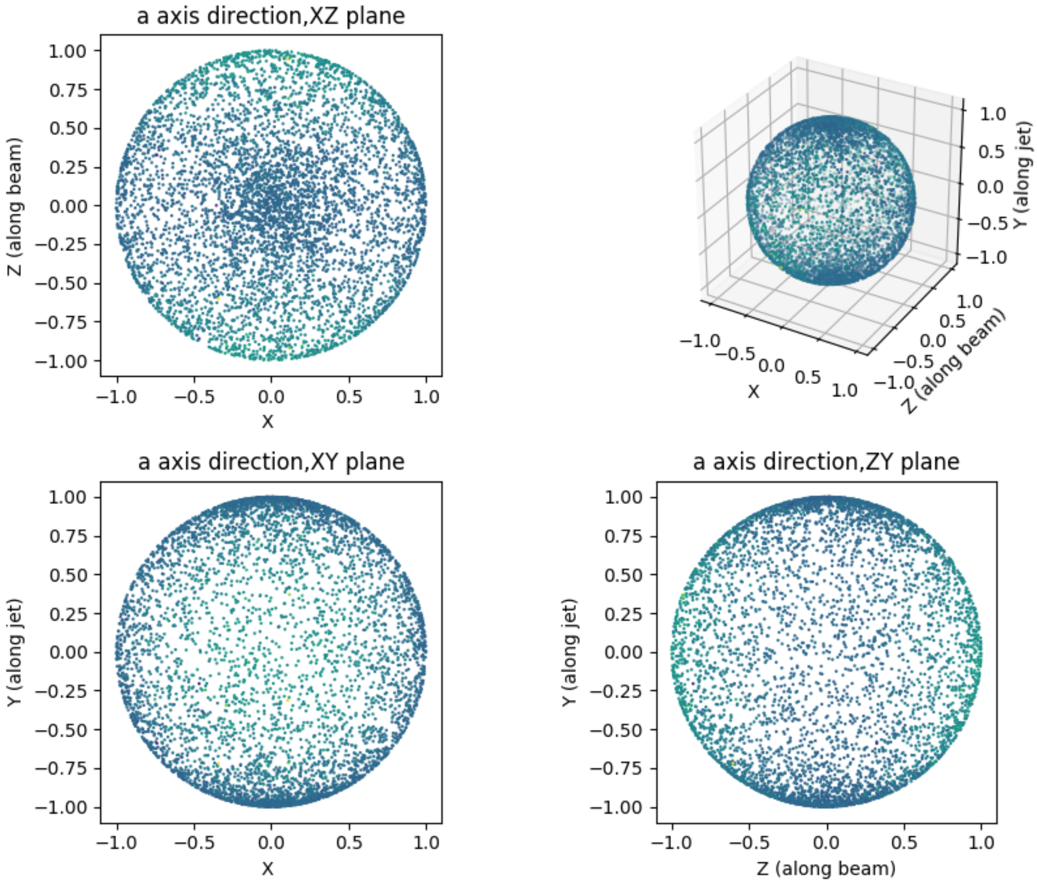

Supplementary Figure 9. **Orientation of the a axis of indexed patterns.** The panels show the orientation of the **a** axis in the xz-, the xy- and zy-plane as well as in a 3D plot. The z-axis is parallel to the X-ray beam axis, the y-axis parallel to the jet axis (right-handed coordinate system). The colour is proportional to the axis length. There is a small preferential alignment of the **a**-axis parallel to the jet axis.

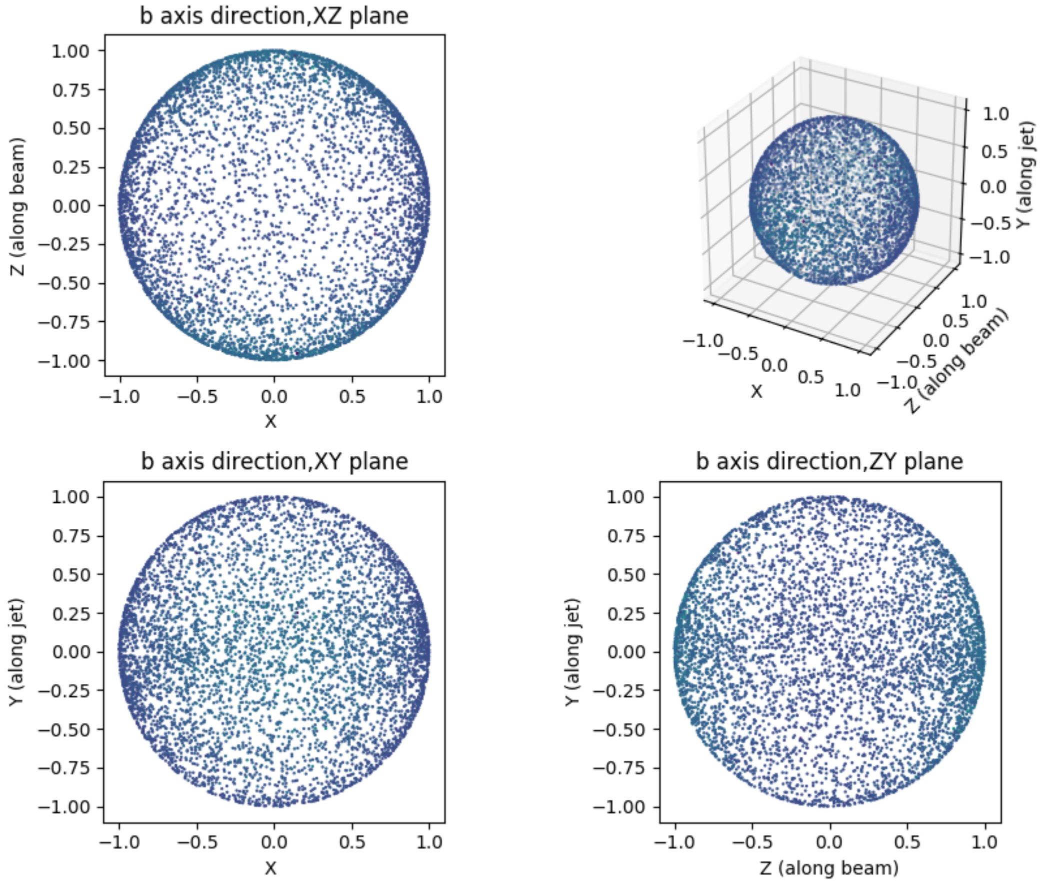

Supplementary Figure 10. **Orientation of the <u>b</u> axis of indexed patterns.** The panels show the orientation of the **b** axis in the xz-, the xy- and zy-plane as well as in a 3D plot. The z-axis is parallel to the X-ray beam axis, the y-axis parallel to the jet axis (right-handed coordinate system). The colour is proportional to the axis length. In line with the **a**-axis having a slight preference to be aligned parallel to the jet axis, the **b**-axis has a slight preference to be oriented in a ring around the jet axis.

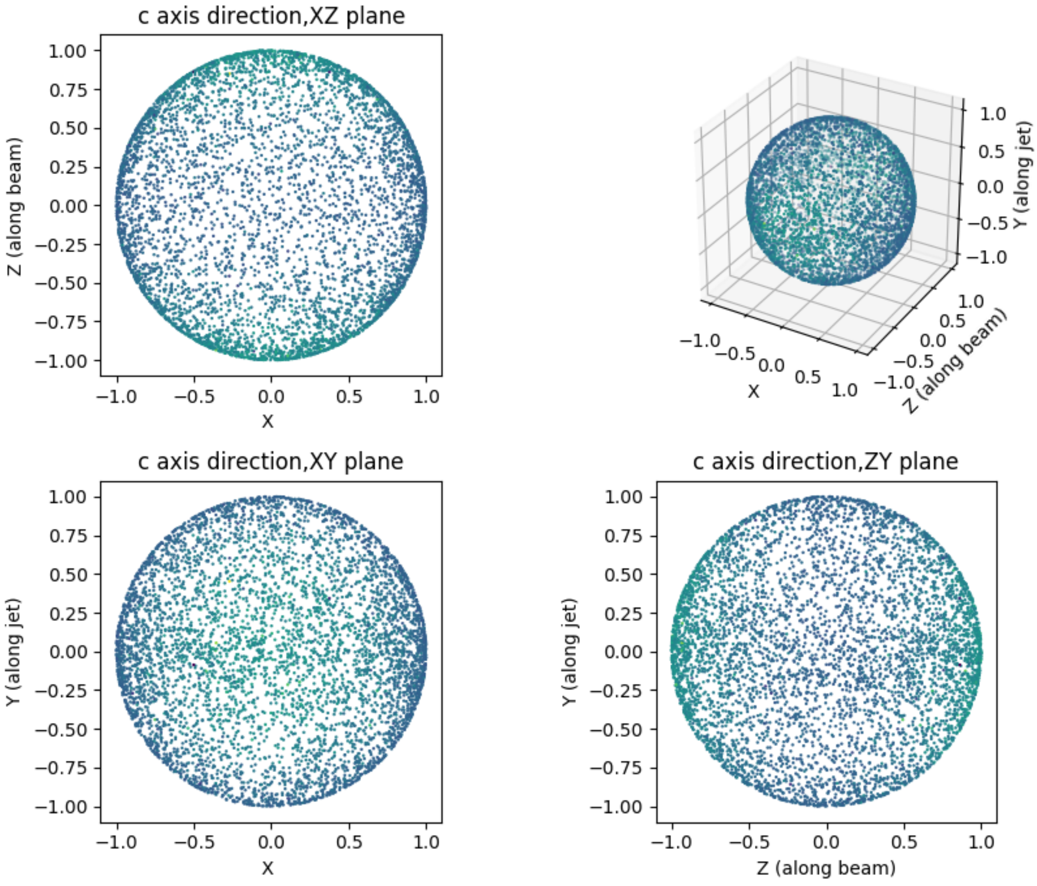

Supplementary Figure 11. **Orientation of the c axis of indexed patterns.** The panels show the orientation of the **c** axis in the xz-, the xy- and zy-plane as well as in a 3D plot. The z-axis is parallel to the X-ray beam axis, the y-axis parallel to the jet axis (right-handed coordinate system). The colour is proportional to the axis length. In line with the **a**-axis having a slight preference to be aligned parallel to the jet axis, the **c**-axis has a slight preference to be oriented in a ring around the jet axis.

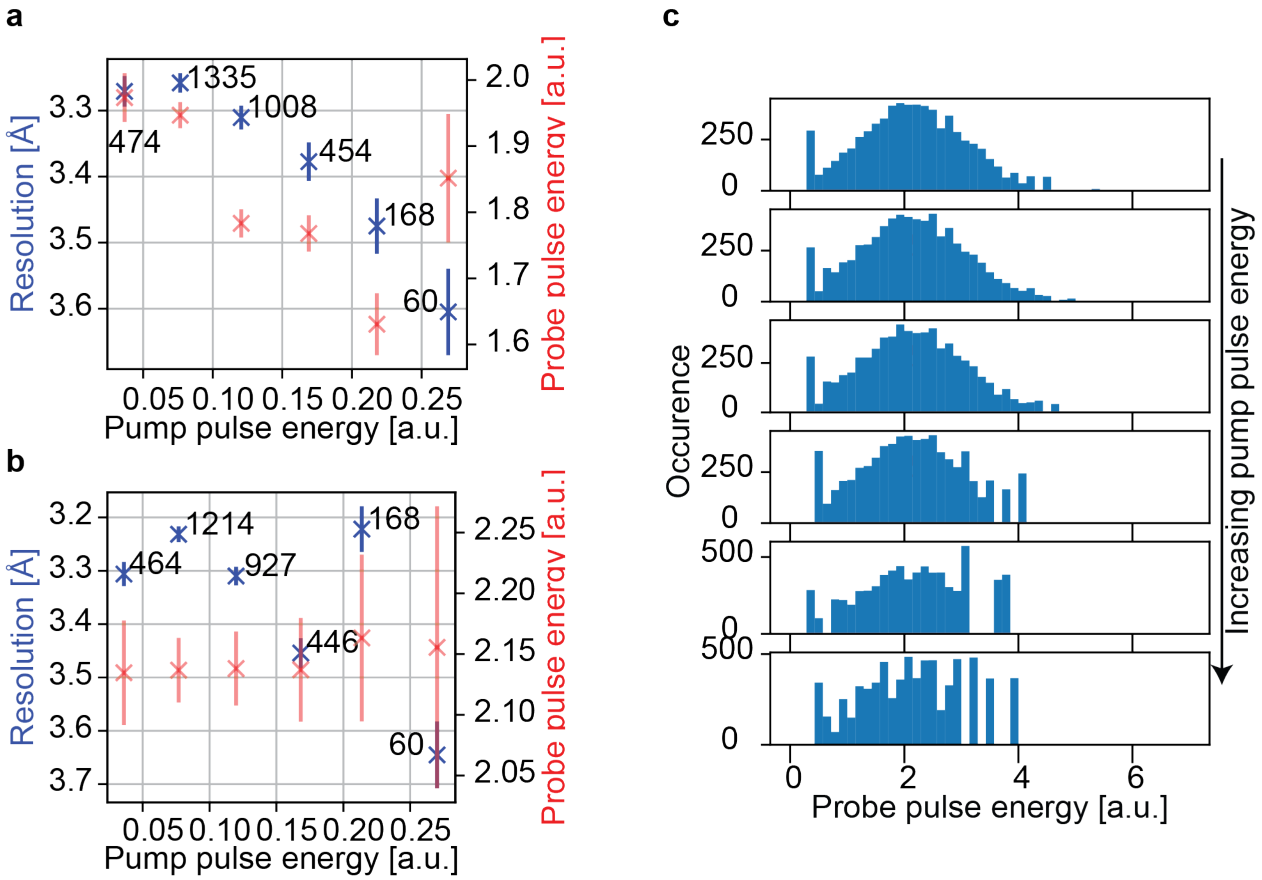

Supplementary Figure 12. **Shock wave damage as a function of shock wave magnitude.** **a**, **b** Diffraction resolution at a signal-to-noise ratio of SNR≥4 (blue, left axis) and probe pulse energy (red, right axis) as a function of pump pulse energy for the pump-probe data set before (**a**) and after (**b**) equalizing probe pulse energies between bins. The number next to each data point indicates the number of unique indexed hits included in each bin. The median resolution of all indexed diffraction images contained in one pump pulse energy bin is plotted. The error bars correspond to the error of the mean per bin. The probe pulse energy was measured by the masked diode. **a** All indexed hits were binned according to the measured pump pulse energy. **b** For six pump pulse energy bins, N=7000 indexed hits were sampled (with replacement) from the corresponding data set to achieve a target probe pulse energy distribution of Gaussian shape with centre μ=2.15 and standard deviation σ=0.94. **c** The probe pulse energy distribution for each pump-pulse energy bin shown in (**b**).

a)

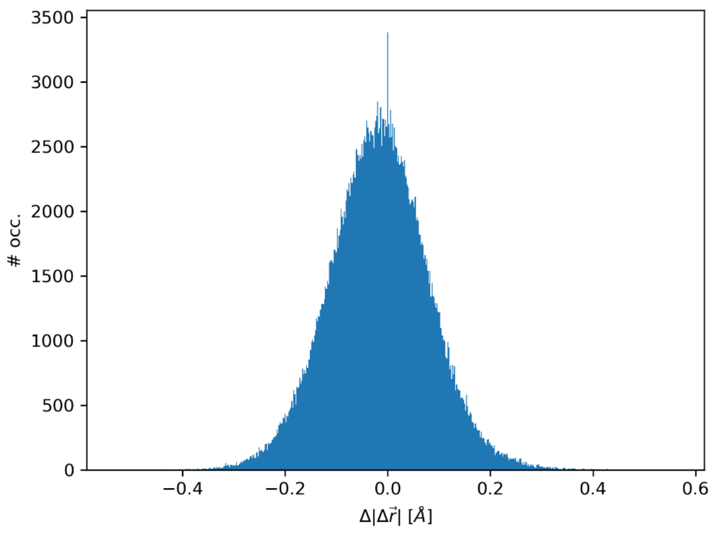

3500
3000
2500
2000
1500
1000
500
0
occ.
−0.4
−0.2
0.0
0.2
0.4
0.6
$\Delta|\Delta\vec{r}|$ [Å]

b)

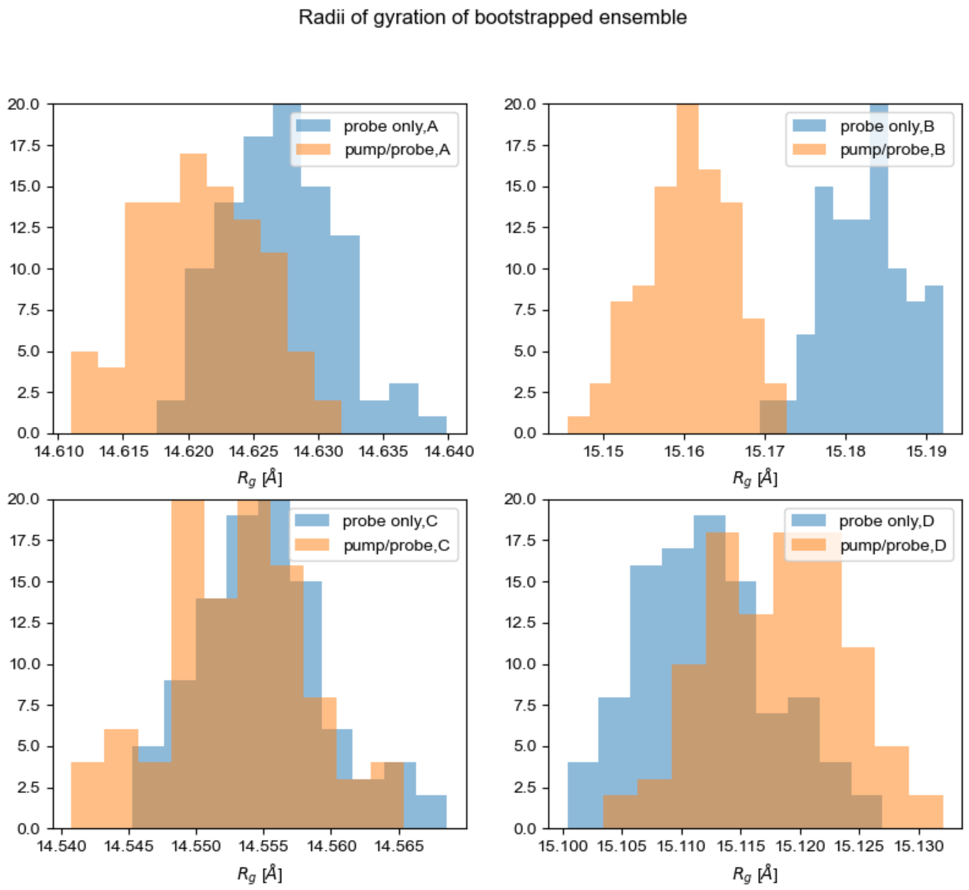

Radii of gyration of bootstrapped ensemble
probe only,A
pump/probe,A
probe only,B
pump/probe,B
probe only,C
pump/probe,C
probe only,D
pump/probe,D
R_g [Å]
14.610
14.615
14.620
14.625
14.630
14.635
14.640
15.15
15.16
15.17
15.18
15.19
14.540
14.545
14.550
14.555
14.560
14.565
15.100
15.105
15.110
15.115
15.120
15.125
15.130

c)

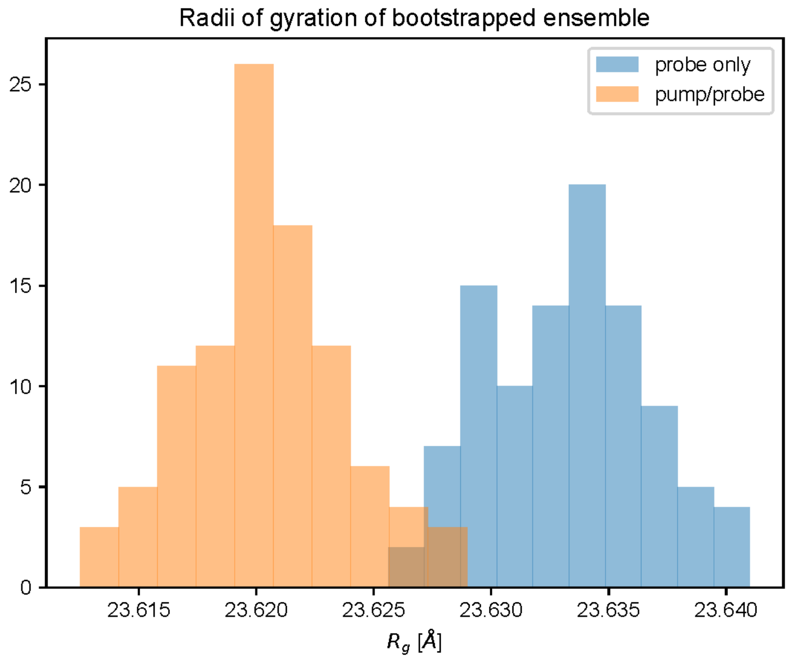

Supplementary Figure 13. Overall structural comparison. a The mean difference of the pairwise distances in the Hb.CO structures determined from the single-pulse and pump-probe data, respectively is slightly negative, indicative of a compaction of the structure in the pump-probe data. Comparison of the radii of gyration of the four HbCO subunits (**b**) and of the tetramer (**c**). Shown are the distributions of the bootstrapped ensembles.

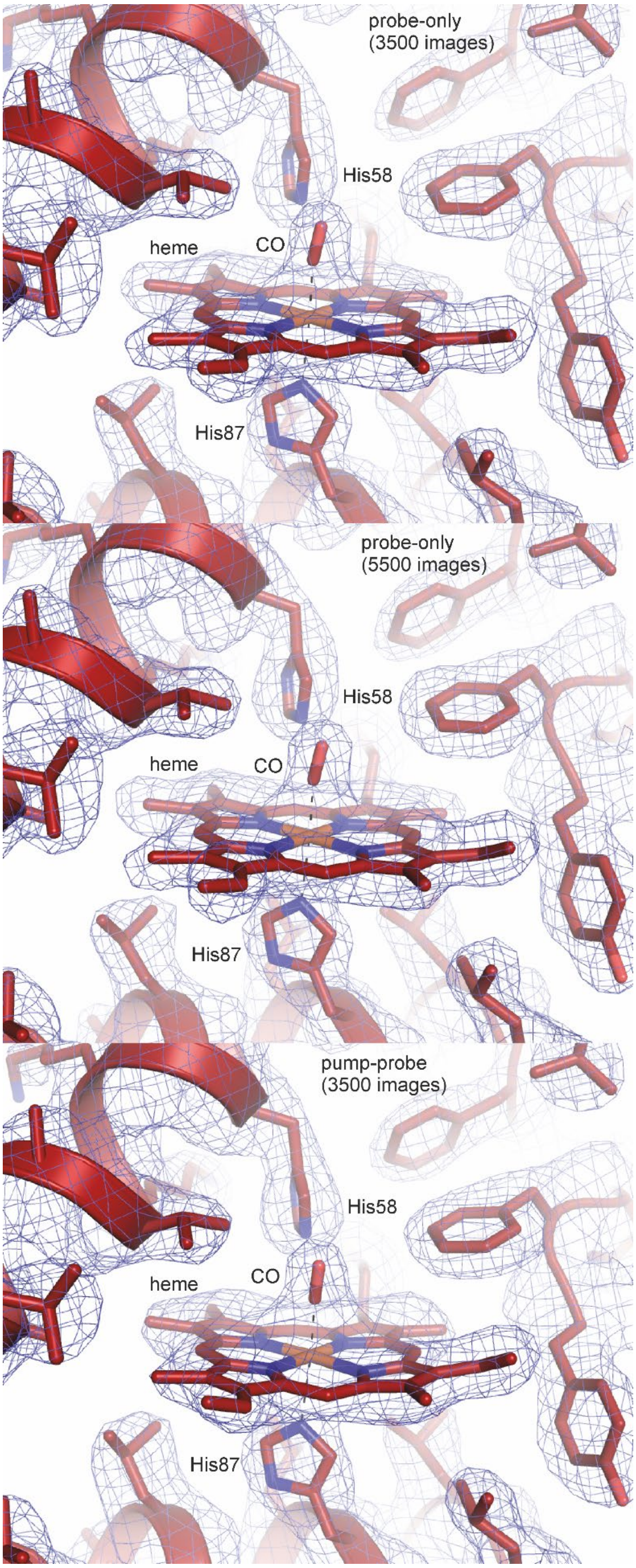

Supplementary Figure 14. Electron density maps around the haem cofactor. 2mFo-DFc electron density maps (1 sigma, blue mesh) overlaid on the final, refined haemoglobin structures in the region around the haem in the A monomer. The structures were derived from the single-pulse data set containing an equal number of images as the pump-probe data set (top), the single-pulse data set including all indexed images (middle) and the pump-probe data set (bottom). For all data sets, only indexed images that passed all filtering steps were included (see also Table 1).

Supplementary Tables

Structure set		Ensemble averaged boot-strapped structures			Single structures, all images used		
Feature		Single-pulse	Pump-probe	Ratio: s-p/p-p	Single-pulse	Pump-probe	Ratio: s-p/p-p
Cavity volume (Å^3)#	Haem A (α1)$	179	160	1.11	177	174	1.01
	Haem B (β1)$	130	129	1.00	129	126	1.02
	Haem C (α2)$	182	168	1.08	182	180	1.01
	Haem D (β2)$	144	148	0.97	141	139	1.01
Packing defects*	RosettaHoles 1.0 score	3.99 (3.56)*	3.32 (3.41)*	1.20 (1.01)*	3.42	3.31	1.03
Secondary structure##	helices	370 (65%)	381 (67%)	0.97	364 (64 %)	388 (68%)	0.93
	Coil	198 (34%)	187 (32%)	1.05	204 (35 %)	180 (31%)	1.13
	turns	28 (4%)	40 (7%)	0.7	32 (5%)	40 (7 %)	0.8

CastP server[1]:

##VADAR server: http://vadar.wishartlab.com/[2]. The secondary structure is identified using three different approaches which are combined yielding a consensus assignment. This explains why values do not add up to 100 %.

*ResProx server: http://www.resprox.ca/[3]. Values in brackets were determined for the ensemble, not the ensemble average

$ Spectroscopic studies have shown shock effects on the haem environment in carbonmonoxy myoglobin[4]; a static high pressure investigation on haemoglobin yielded a much lower compressibility for the α than the β subunits (κ_α: 0.17±0.05 GPa^{-1} versus κ_β: 0.36±0.07 GPa^{-1})[5]. Our structural analysis of the haem binding cavities shows no difference in the volume of the haem cavities determined from the single-pulse and pump-probe data, respectively. The comparison of the ensemble-averaged structures indicates a contraction of the α-cavities in the pump-probe data compared to the single-pulse data and no significant changes in the β subunits.

Supplementary Table 1. Features of the single-pulse and pump-probe data.

Supplementary Note 1

Equivalence of the pump-probe setup and a 4.5 MHz experiment

The described experiment was performed at a pump-probe time delay of 122.5 ns, mathematically corresponding to a repetition rate of ~8 MHz. However, due to the vertical offset of ~5 µm between the pump and probe pulse, the segment probed by the X-ray probe would have been in the pump interaction region at a later time point. Given that the jet speed was ~50 ± 5 m/s, it took the jet ~100 ns to cover the 5 µm distance between pump and probe interaction region. Thus, the jet segment probed by the second X-ray pulse would have been in the pump interaction region after 122.5 + 100 ns = 222.5 ns, corresponding to a repetition rate of ~4.5 MHz for pulses focused on the same interaction point.

Supplementary Note 2

Apparent unit cell shift between pump probe and probe only data set

A small shift (≤0.5 %) in unit cell constants is observed between the pump-probe and the probe-only data set, with the unit cell apparently shrinking for the pump-probe data collection. This can be explained by a difference in the value of the X-ray wavelength used by the indexing program for the two data sets.

To account for shot-to-shot variations in the average photon energy of a XFEL pulse, which is a normal feature of the self-amplified stimulated emission (SASE) operation, the indexing program extracts for each diffraction image the X-ray wavelength that is estimated from the properties of the electron bunches in the accelerator. In the single-pulse data, the photon energy is properly measured, and was targeted to ~7.07 keV (i.e., ~40 eV below the Fe absorption edge at 7.11 keV). However, due to the short temporal separation of the two pulses used in the pump-probe data, the average photon energy of the pump and probe pulses (differing by ~80 eV) is measured and this value is written into the image header instead of the real value of the probe pulse photon energy used for acquisition of the pump-probe data (nominally ~7.07 keV). The erroneous average value is then used by CrystFEL. Thus, in the pump-probe runs the indexing program assumes a photon energy that is ~0.5% higher than its nominal value, which results in estimates of the unit cell axis that are ~0.5% smaller than their true values. Therefore, the ~0.5% smaller unit cells of the pump-probe correspond to

the unit cells in the single-pulse data when taking into account the systematic error introduced by using the facility-supplied values of the average photon energy in two-bunch mode. This rational assumes that both the pump energy distribution and the pulse energy separation, respectively, of the two pulses remains the same during the experiment. Both can change during the course of the experiment and either would affect the scaling ratio between the unit cell constants. All three cell length would be affected the same way. We observe slightly different ratios for the three unit cell lengths (reduction of 0.4 %, 0.3 %, 0.5 % of the pump-probe unit cell lengths *a*, *b*, *c*, respectively, compared to the single-pulse values). This difference in axis length reduction could be due – at least partially - to indexing uncertainties. Indeed, due to flow alignment of the Hb.CO microcrystals in the liquid jet, the ***a*** axis is oriented preferentially parallel to the jet axis (Supplementary Figure 9), resulting in a larger error in determining the lengths of *b* and *c* axis. It is. In addition, pressure-induced changes of one or two unit cell constants are conceivable.

Supplementary Note 3

Experimental geometry probes shock wave damage and not radiation damage

The combination of our experimental geometry (X-ray pump and probe location, beam sizes, crystal size) with the time between X-ray pulses and jet speed was chosen such as to exclude radiation damage and ensure that the observed changes are due to shock wave damage. There are two scenarios how the first pulse could inflict radiation damage on sample probed with the second pulse:

1.) Stray light from pump pulse affects the site of the crystal going to be interrogated by the probe pulse; or
2.) Photoelectrons and/or radicals created by first pulse diffuse to the site interrogated by the probe pulse.

Given the setup of the experiment, the probability is extremely low for both scenarios.

The pump and probe X-ray beams (Ø ~1.5 µm (FWHM) each) are separated by 5 µm, moreover, the jet (including the crystals) moves ~ 6 µm during the 122.5 ns time delay between the two pulses (jet speed 50 m/s). Thus, the distance between the first pump XFEL shot and

the second probe XFEL shot along the jet is ~ 11 µm, which is longer than the longest crystal dimension (10 µm). Therefore, a crystal being hit with its front end centred on the pump pulse is thus located 1 µm below the probe pulse interaction region at the time of the probe pulse arrival (Supplementary Figure 7, 8, Supplementary Software). Thus, damage can only be induced either directly by the "wings" of the focused pump beam or indirectly by photoelectrons or diffusing radicals. The latter are too slow to reach the upstream end of the crystal before the probe beam hits, 122.5 ns after the first pulse. The free path length of ~12 keV photoelectrons is on the order of 6 µm (see references[6,7]); it is shorter for ~ 7 keV photoelectrons (our experiment). So they too are highly unlikely to cause damage. Assuming a Gaussian beam profile, the intensity of the FEL wings at the probe position (10 µm upstream of the pump pulse) is $3 \cdot 10^{-52}$ of that of the pump beam at the focal position and thus negligible. Therefore, also the "stray" X-ray photons are extremely unlikely to induce any measurable damage.

Moreover, the situation described above applies only to crystals that are oriented with their longest axis parallel to the jet axis. While flow alignment can be detected in the indexed data (Supplementary Figure 9-11), it is not dominant. Thus, most crystal hits have a much shorter length along the jet axis, making, radiation damage due to double hits even more unlikely (Supplementary Figure 7).

In conclusion, the observed damage cannot be radiation damage but must be due to shock damage, an effect that can overcome longer distances.

Supplementary Note 4

Analysing damage as a function of pump pulse energy

Since the magnitude of the launched shock wave increases with the energy of the pump pulse, the dependence of the shock wave damage on the shock pressure will be similar to the dependence of the damage on the pump pulse energy. To compare the diffraction quality of data sets with different pump pulse energies, the distribution of probe pulse energies should be similar between subsets. However, in the two-bunch mode the tuning of the pulse energy of one pulse can affect the pulse energy of the other pulse, leading to possible correlations

of the two pulse energies. In our data an anti-correlation between pump and probe pulse energy was observed. This does not allow to differentiate whether the observed decrease in resolution as a function of pump pulse energy is caused by shock wave degradation or by the decrease in probe pulse energy (Supplementary Figure 7a).

Therefore, equalization of probe pulse energy to similar distributions was performed for all pump energy bins as described in (Supplementary Methods 2). Although after equalization the data for the highest pump energies had a lower resolution, it cannot be stated with confidence that the degree of damage increases with the pump energy (Supplementary Figure 7b): Due to the generally small number of indexed hits in the data set and due to the strong correlation between pump and probe pulse energy, too few hits have comparable probe pulse energies leading to a bad sampling of probe pulse energies especially at the highest pump pulse energies, where an increase of damage is expected (Supplementary Figure 7c).

Supplementary Note 5

Comparison of shock pressure between our experiment and a previous experiment at 1.1 MHz

Previous to this publication, the most severe (because of a tight ~ 2-3 µm X-ray focus) conditions tested for shock damage were those by Yefanov *et al.*[8], where SFX data was collected on lysozyme at 1.1 MHz repetition rate using X-ray pulses with a pulse energy of 0.8 mJ/pulse on average at the sample position and employing liquid jets of 100 m/s speed and 2-3 µm diameter for sample delivery. No indication for shock-induced damage was found in this analysis[8].

We estimate the ratio of shock pressures in the our experiment as well as in the one by Yefanov *et al.* [8], from here on referred to as the Yefanov EuXFEL experiment, using their median experimental parameters. Our experiment used 0.03 mJ pulses at 7.1 keV in a 5-µm diameter jet, and the pump and probe pulses were separated by 11 µm along the jet (Supplementary Note 1). The Yefanov EuXFEL experiment used 0.8 mJ pulses at 9.3 keV in a 2.5-µm jet, and consecutive pulses were separated 89 µm along the jet (based on the pulse rate and the jet velocity). Following Blaj *et al.*[9], the pressure of the initial cylindrical shock launched by the XFEL is approximately proportional to the energy that the pulse deposits

into the jet, which depends both on the pulse energy and its absorption. Before starting to propagate in the jet, the shock must propagate cylindrically until it "fills" the jet, and we approximate that the pressure scales inversely proportional to the shock radius (this scaling approximately conserves the compressive energy in a cylindrical shell). Considering that the X-ray absorption in water at 9.3 keV is approximately half of that at 7.1 keV, the pressure of the EuXFEL shock (when it starts to propagate in the jet) is larger than the one in our experiment by a factor of (0.8 mJ / 0.03 mJ)×0.5×(5 µm / 2.5 µm) ≈ 27. Following the same scaling procedure against the measurements reported in Blaj *et al.*[9], we also estimate that the initial shock pressure was on the order of a few hundred MPa in our experiment, and on the order of several GPa in the Yefanov EuXFEL experiment.

Although the initial shock pressure is larger in the Yefanov EuXFEL experiment, it will decay more due to the longer distance of propagation. Studies in larger diameter water jets[9,10] showed that the pressure decay is initially very rapid, but after reaching a peak pressure of ≈40 MPa it slows down. It was proposed that this subsequent slower decay is due to ultrasonic attenuation. In our experiment, the shocks travel a distance of approximately two jet diameters before reaching the sample, which is a regime in which the initial rapid decay (one order of magnitude in pressure for a propagation distance equal to 1.5 jet diameters) is the dominant mechanism. Given the initial pressure on the order of a few hundred MPa, we estimate that in our experiment the shocks had a pressure in the vicinity of the ≈40 MPa pressure level where the pressure decay rate starts to slow down. In the EuXFEL experiment, the ≈40 MPa pressure level was reached after the shock travelled approximately 3–4 jet diameters (≈8–10 µm), and then the shock continued to decay slower, through ultrasonic attenuation, until it reached the next sample. Therefore, the shock pressure at the sample in the Yefanov EuXFEL experiment is smaller than in our experiment by the ultrasonic attenuation decay factor for ≈ 89−9 = 80 µm of propagation in the 2.5-µm diameter jet. To estimate the ultrasonic attenuation, we expressed the exponential attenuation length in water, $\alpha_f = 2.53\times10^{-14}\ f^2$ where f is the sound frequency, as a function of the frequency associated with the shock train $f_S = c_0/R$, where c_0 is the speed of sound and R is the jet radius. This leads to an exponential attenuation length of $\alpha_f = 5.69\times10^{-8}\ R^{-2}$, and to an ultrasonic attenuation factor of ≈18 for the Yefanov EuXFEL experiment. Therefore, we estimate that the shock pressure in the Yefanov EuXFEL experiment was on the order of a few MPa, and at least one order of magnitude smaller than in our experiment. Our observation of a shock-

induced structural change is thus compatible with the lack of shock-induced effects observed in the 1.1 MHz Yefanov EuXFEL experiment due to the large difference in the shock pressures at the sample.

Supplementary Note 6

Notes on performing shock-damage experiments

The most straightforward way to reduce the shock wave pressure, and thus the damage, is to lower the pulse energy, but this approach has the disadvantage that it concomitantly reduces the diffraction intensity and therefore the observable scattering resolution. Also, the entanglement of pressure-jump related effects and probe-intensity related effects will complicate the analysis. Instead, future MHz-(SFX) measurements investigating pressure-jump effects can vary the pulse repetition rate, the jet velocity, or the jet diameter to change the pressure of the shock wave. Lowering the X-ray repetition rate will increase the separation between the hit regions along the jet, allowing the shock to decay more before it reaches subsequent samples. Smaller diameter jets decrease the decay length of the shock wave, which provides a different mechanism to reduce the pressure of the shock at the subsequent samples[9]. In faster jets the jet segment that passes between two shots is longer, increasing the absolute length along which the shock decays.

Supplementary Methods

Filtering jet images

Femtosecond snapshot images of the jet were recorded a few nanoseconds after impact of the probe pulse onto the jet. Based on the shape of the jet, on the visible gaps in the jet that were created by XFEL-induced explosions, and on other visible gaps in the jet (e.g. due to droplet break-up), hits can be filtered according to whether the pump pulse launched a shock wave affecting the sample segment interrogated by the X-ray probe pulse.

Filtering of the jet images was performed in three steps: (i) The shape of the jet was analysed to determine whether the jet was much thinner or thicker or differed otherwise from the typical appearance in which case the corresponding hit was discarded (Supplementary Figure 1a). (ii) Gaps inside the jet were identified and their location and size determined, analysing the jet region from ~10 µm downstream of the pump interaction region to ~22 µm upstream of the probe interaction region. (iii) The number and location of the gaps was studied to identify images with jets having more than 2 gaps (jet had likely broken up, prohibiting shock wave propagation), images with jets having no gaps (pump pulse had likely not hit the jet and thus did not launch a shock wave) and images of jets where at least one of the identified gaps was less than ~4.5 µm upstream of the probe interaction region (probe pulse may have probed a droplet not affected by shock waves) (Supplementary Figure 1b,c). In all these cases, the corresponding hit was discarded.

For analysing jet shape (i) the projected size of the jet was determined from images of the jet (Supplementary Figure 2a) by calculating the mean pixel intensity of pixel columns parallel to the jet axis (i.e. projecting pixel intensities onto the horizontal (x-)axis). The projected jet size was defined as the 'full-width half minimum' region in which the mean projected intensity was smaller than a threshold defined by the median intensity minus half the difference between the median and minimum intensity of the projection (Supplementary Figure 2b). Discarding hits with projected jet sizes deviating by more than one standard deviation from the median projected jet size ensures that jets of abnormal shape (e.g. those that are extremely thin and about to break, extremely thick after resolution of a clog, extremely wiggly, …) are not part of the shock damage analysis since shock wave propagation in such jets differs from shock wave propagation in standard straight column jets (Supplementary Figure 1).

Next, gaps in the jet were evaluated (ii) based on the median filtered jet images, obtained using a median filter of 5x5 pixels to smoothen the remaining speckle pattern in the background. The region of interest containing the jet was evaluated from the projected jet size plus a buffer of 11 µm to the left and right of the jet centre (red rectangle in Supplementary Figure 2a). For each row of pixels perpendicular to the jet axis it was determined whether it contains a segment of the jet by comparing the minimum pixel intensity of the background to the minimum pixel intensity inside the region of interest containing the jet (Supplementary Figure 2c,d). If pixel intensities inside the region of interest decreased below 90 % of the minimum pixel intensity of the background, a jet segment is seen in a given column row (Supplementary Figure 2c), otherwise a given row of pixels is defined as representing a gap in the jet (Supplementary Figure 2d). After iterating through each row of pixels in the image (running perpendicularly to the jet axis) to decrease vulnerability of the algorithm to noise, gaps closer than 2 pixels to each other were merged and only gaps of at least two pixels in size were considered. To identify number and location of gaps (iii) connected gap regions (i.e. neighbouring rows considered as a ‘gap’ in the jet) were counted and their extent measured.

Sampling data subsets with equalized probe pulse energies

Diffraction quality not only depends on crystalline order, which may be altered by the impact of shock waves, but scales also with the pulse energy of the probing X-ray pulse. The distribution of probe pulse energies was therefore compared between pump-probe and single-pulse data sets which revealed a difference of 14 % in the average probe pulse energy (Supplementary Figure 4b). To exclude probe pulse energy changes impacting diffraction resolution, subsets of each data set were created in which the distribution of probe pulse energies was equalized.

For two existing data sets A and B having given distributions in probe pulse energy with mean μ_A and μ_B and standard deviations σ_A and σ_B the target probe pulse energy distribution was determined as a Gaussian distribution centred on $\mu=0.5\cdot(\mu_A + \mu_B)$ with standard deviation $\sigma=0.5\cdot(\sigma_A + \sigma_B)$. From this distribution N random probe pulse energies e were sampled.

To form two subsets A* and B* with equal probe pulse energy distributions, all sampled probe pulse energies *e* were compared to the probe pulse energies in the original data sets in A and B, and the hit with the closest probe pulse energies in each of A and B, respectively, was added to the requisite subsets A* and B*.

Supplementary References

1. Tian, W., Chen, C., Lei, X., Zhao, J. & Liang, J. CASTp 3.0: computed atlas of surface topography of proteins. *Nucleic Acids Research* **46**, W363-W367 (2018).
2. Willard, L. *et al.* VADAR: a web server for quantitative evaluation of protein structure quality. *Nucleic Acids Research* **31**, 3316-9 (2003).
3. Berjanskii, M., Zhou, J., Liang, Y., Lin, G. & Wishart, D.S. Resolution-by-proxy: a simple measure for assessing and comparing the overall quality of NMR protein structures. *Journal of Biomolecular NMR* **53**, 167-80 (2012).
4. Dlott, D.D. Ultrafast spectroscopy of shock waves in molecular materials. *Annual Review of Physical Chemistry* **50**, 251-78 (1999).
5. Schay, G. *et al.* Dissimilar flexibility of alpha and beta subunits of human adult hemoglobin influences the protein dynamics and its alteration induced by allosteric effectors. *PLoS One* **13**, e0194994 (2018).
6. Nave, C. & Hill, M.A. Will reduced radiation damage occur with very small crystals? *Journal of Synchrotron Radiation* **12**, 299-303 (2005).
7. Sanishvili, R. *et al.* Radiation damage in protein crystals is reduced with a micron-sized X-ray beam. *Proceedings of the National Academy of Sciences* **108**, 6127-6132 (2011).
8. Yefanov, O. *et al.* Evaluation of serial crystallographic structure determination within megahertz pulse trains. *Struct. Dynamics* **6**, 064702 (2019).
9. Blaj, G. *et al.* Generation of high-intensity ultrasound through shock propagation in liquid jets. *Phys. Rev. Fluids* **4**, 043401 (2019).
10. Ursescu, D. *et al.* Generation of shock trains in free liquid jets with a nanosecond green laser. *Physical Review Fluids* **5**, 123402 (2020).
