# Supplementary material for: Effect of X-ray free-electron laser-induced shockwaves on haemoglobin microcrystals delivered in a liquid jet
Source: Nat Commun. 2021 Mar 15;12:1672. doi: 10.1038/s41467-021-21819-8 (PMC7960726; doi:10.1038/s41467-021-21819-8)
Supplement: Supplementary file 3 — Description of Additional Supplementary Files [file 41467_2021_21819_MOESM3_ESM.pdf]

Description of Additional Supplementary Files

Supplementary Movie 1: Movie of consecutive pump-probe pairs hitting the jet. Consecutive pump-probe pairs hit the jet, leading to distinct jet explosions at the interaction regions. The two gaps are at a distance of ~11 µm to each other, originating from 5 µm vertical offset between pump and probe interaction region and ~6 µm jet translation between pump and probe pulse arrival. The jet instabilities (wiggling) are due to the high crystal concentration used in our experiment; generally, jet instabilities correlate with crystal concentration.

Supplementary Movie 2: Structural comparison of the single-pulse and pump-probeHb.CO structures. The displacement between respective Cα positions is indicated by black arrows. The magnitude of the displacement is illustrated by the length of arrows (multiplied by a factor of 10).

Supplementary Movie 3: Structural comparison of the single-pulse and pump-probe Hb.CO structures. The displacement between respective Cα positions is indicated by black arrows. The magnitude of the displacement is illustrated by the length of arrows (multiplied by a factor of 10). The orientation of the tetramer differs by a 90°rotation from the one in Supplementary Movie 2.

Supplementary Movie 4: Structural comparison of the single-pulse and pump-probe Hb.CO structures. The movie shows a morph between the structures determined from the single-pulse and pump-probe data, respectively. The differences are amplified by a factor of 4.

Supplementary Movie 5: Analysis of channels in the Hb.CO structures. Channels identified in the structures determined from the single-pulse (pink) and pump-probe (lemon) data, respectively. Cavities and channels were identified using radii for the outer and inner probe of 6 and 1.5Å, respectively (see Voss, N.R. & Gerstein, M. 3V: cavity, channel and cleft volume calculator and extractor. Nucleic Acids Res 38, W555- 562 (2010)).

Supplementary Software 1: The spreadsheet allows to calculate the probability of consecutive (dual) XFEL hits of the same crystal as a function of crystal length and jet speed. While it was conceived for the X-ray pump X-ray probe experiment described in this manuscript, it is a useful visualization tool for MHz data collection in general.
